# Investigation of Superhydrophobic and Anticorrosive Epoxy Films with Al_2_O_3_ Nanoparticles on Different Surfaces

**DOI:** 10.1021/acsomega.3c00729

**Published:** 2023-06-06

**Authors:** Merve Dandan Doganci, Hakan Sevinç

**Affiliations:** † Polymer Science and Technology Graduate Program, Kocaeli University, Kocaeli 41380, Turkey; ‡ Department of Chemistry and Chemical Processing Tech., Kocaeli University, Kocaeli 41140, Turkey

## Abstract

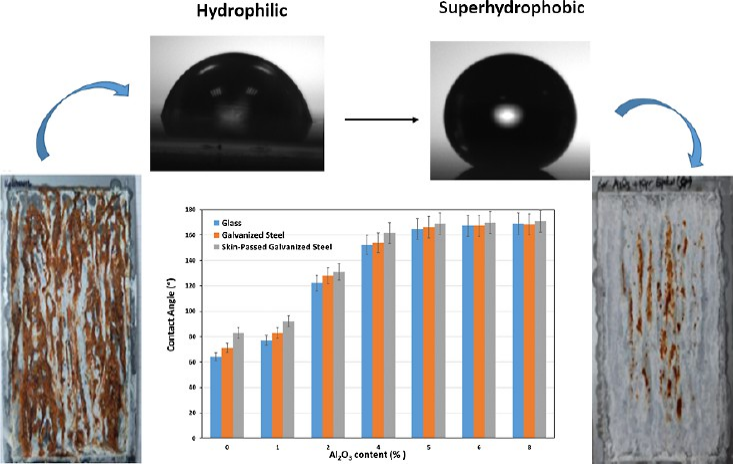

In this study, superhydrophobic epoxy coatings were prepared on different surfaces by utilizing hydrophobized aluminum oxide (Al_2_O_3_) nanoparticles. The dispersions containing epoxy and inorganic nanoparticles with different contents were coated on glass, galvanized steel, and skin-passed galvanized steel substrates by the dip coating method. The contact angles of the obtained surfaces were measured via a contact angle meter device, and the surface morphologies were analyzed by utilizing scanning electron microscopy (SEM). The corrosion resistance was performed in the corrosion cabinet. The surfaces showed superhydrophobic properties with high contact angles greater than 150° and self-cleaning properties. SEM images indicated that the surface roughness increased as the concentration increased by the incorporation of Al_2_O_3_ nanoparticles into epoxy surfaces. The increase in surface roughness was supported by atomic force microscopy analysis on glass surfaces. It was determined that the corrosion resistance of the galvanized and skin-passed galvanized surfaces increased with the increase of Al_2_O_3_ nanoparticle concentration. It has been shown that red rust formation on skin-passed galvanized surfaces was reduced, although they have low corrosion resistance due to roughening on their surfaces.

## 1 Introduction

Superhydrophobic surfaces are having great interest due to their unique characteristics like self-cleaning,^1−5^ anti-sticking,^6^ anti-corrosion^4,7−10^ etc. in many industrial applications. There are mainly two basic procedures to design superhydrophobic surfaces. The first procedure is to produce hierarchical micro–nano roughness on the hydrophobic surfaces, and the other one is to make modifications to the rough surface to lower the surface free energy using silicones, fluorocarbons etc.^11−13^ In recent years, the addition of inorganic metal oxide nanoparticles like SiO_2_, TiO_2_, Al_2_O_3_ etc. to polymeric matrix is an easy and practical method to create multi-scale roughness to enhance the superhydrophobicity.^14−17^ Alumina (Al_2_O_3_) nanoparticles are one of the important materials that are utilized for creating superhydrophobic surfaces.^14,18−25^ Barron et al. used unmodified and chemically modified alumina nanoparticles. They used the carboxylic acids for modification on nanoparticles and investigated the effects of carbon chain lengths and branching factors on the surface modification of nanoparticles. The hydrophobicity and oleophobicity were increased by introducing branches and chain functionality into the system.^26−28^ Sutha and co-workers prepared transparent superhydrophobic surfaces on glass surface with a contact angle of 161° to be used on solar panel applications.^22^ Karapanagiotis et al. studied with different nanoparticles, including hydrophilic alumina nanoparticles, and showed that superhydrophobicity could be achieved by using nanoparticles having different particle sizes. They also concluded that the particle size was not important on the hydrophobicity of the surfaces, but the concentration had a significant impact.^14,19,29^ However, Richard and co-workers also used different particle-sized alumina that were modified with stearic acid. They obtained superhydrophobic glass and cotton fabric surfaces having self-cleaning properties with 0.30 μm alumina micro particles.^23^ Sutar et al. coated glass surfaces with alumina, polymethylhydrosiloxane (PMHS), and polystyrene (PS) by dip-coating technique and obtained stable superhydrophobic surfaces.^24^ Zhang and co-workers created superamphiphobic surfaces by utilizing polymerized organosilanes and Al_2_O_3_ nanoparticles.^21^ Ji et al. investigated the effects of the content of alumina and PDMS on the contact angle of cotton fabrics. The obtained fabrics had excellent superhydrophobicity and self-cleaning properties.^25^

The fabrication of superhydrophobic epoxy surfaces is of great interest to use epoxy coatings as water-repellent surfaces.^5,18,30−33^ Penna et al. modified the alumina nanoparticles (NPs) with stearic acid in two different solvents (2-propanol and toluene), then applied them on two different epoxies by bilayer coating in two steps; diglycidyl ether of bisphenol A (DGEBA) or Novolac Type II resin; to prepare superhydrophobic epoxy surfaces. They observed a superhydrophobic surface with roll of water drops on a DGEBA-based coating having functionalized alumina NPs in 2-propanol.^18^ Wu et al. obtained superhydrophobic epoxy coating with alumina nanoparticles with a different method that is an inverse infusion process.^33^ Zhong et al. fabricated transparent superhydrophobic epoxy coatings, including hydrophobic silica nanoparticles.^30^ Alamri and co-workers created magnetic superhydrophobic epoxy surfaces with superhydrophobic silica-coated magnetite nanoparticles. They achieved a contact angle of 175° on the surfaces with good stability and good durability under different corrosive conditions.^31^

Metals are highly corrosive materials and generally used by galvanizing (coating with molten zinc). The galvanized steels are widely used in many products and sectors, such as automobiles, white goods, insulation and roofing, pipes, water and fuel tanks, package etc.^34−39^ The formation of zinc compounds called white rust is observed on the surfaces as a result of corrosion of the upper zinc coating on galvanized steel surfaces. The white rust progresses after a while, and red rust formation begins on the steel layer.^35,39^ If the surface was designed to be painted, the roughening process (skin-pass) is applied to metal surfaces. This procedure is used to increase the surface area and adhesion of the organic coating, but it decreases the corrosion resistance of the zinc coating.^40^ Epoxy resins are one of the coatings that are used for corrosion prevention by acting as a physical-barrier.^41−44^ Yu et al. used alumina particles to improve the anti-corrosive character of epoxy films and compared the effects of hydrophilic and superhydrophobic alumina particles. They concluded that the incorporation of silane-functionalized alumina improved the corrosion resistance of epoxy resins in high salinity solution.^43^ Samardžija et al. used epoxy modified with Al nanoparticles to protect the surface of gray cast iron from corrosion. They concluded that addition of 0.75% Al in the coatings gave the best corrosion resistance.^44^

In this work, superhydrophobic epoxy films were gained on different surfaces by utilizing commercially hydrophobized aluminum oxide (Al_2_O_3_) nanoparticles. The dispersions, including epoxy and inorganic nanoparticles with different contents, were coated on glass, galvanized steel, and skin-passed galvanized steel substrates by an easy, reliable, and low-cost dip coating method. It is one of the most common industrial film fabrication process allowing to prepare surfaces with multi-component systems in one-step and to apply any size of surface having a uniform thin film morphology. The Attention Theta Lite contact angle meter device was utilized for determining contact angles of the substrates, and the surface morphologies of the obtained superhydrophobic surfaces were determined via scanning electron microscopy (SEM). The corrosion behavior was determined by testing the surfaces in the corrosion cabinet. The finally prepared surfaces showed superhydrophobic properties with a contact angle greater than 150°.

## 2 Experimental Section

### 2.1 Materials

Epoxy resin based on diglycidyl ether of bisphenol-A (EPON 1001-X-75, epoxy equivalent 450–550 g/equiv) and methylated high-imino melamine formaldehyde (Cymel 325) were supplied by DYO. Highly dispersed hydrophobic fumed aluminum oxide, Aeroxide Alu C 805 (average primary particle size: 13 nm), was kindly provided from Evonik Industries. It is a fine particulate powder having specific BET surface area of 75–105 m^2^/g with an alumina content of ≥95%. It is hydrophobized by treating with octylsilane. The solvents, such as toluene and pure water, were purchased from Merck.

#### 2.1.1 Substrate Specimens

Three different substrates were used for coating: glass slides, galvanized steel, and skin-passed galvanized steel surfaces. Glass slides with a dimension of 76 × 26 mm were cleaned by using acetone and pure water, dried at 100 °C for 2 h, and kept in a desiccator before using. The chemical compositions of the steel surfaces are presented in Table S1. One of the main purposes of surface transition rolling is to obtain a certain surface roughness profile. The surface roughness of the steel surface affects the corrosion resistance, appearance after painting, and formability in press forming. The skin-pass process is applied to surfaces before organic coating to increase the surface area of the galvanized sheet. Thus, the organic coating adheres to the surface better. The steel surfaces were cleaned with isopropyl alcohol, dried in the oven, and held in desiccators prior to use.

### 2.2 Preparation of Superhydrophobic Epoxy Surfaces

**Scheme 1:**
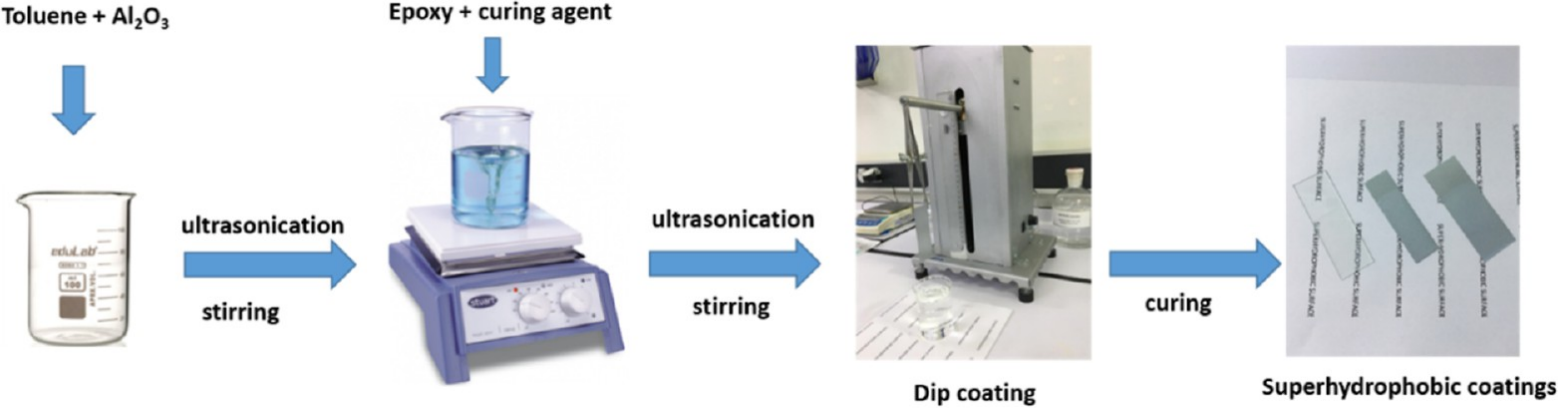
Preparation of Superhydrophobic Epoxy/Alumina Surfaces

10 g of epoxy polymer having its curing agent was dissolved in toluene, and Al_2_O_3_ nanoparticles were dissolved separately using an ultrasonic bath (DAIHAN WUC-A03 Analog Ultrasonic Cleaners) at different concentrations (1, 2, 4, 5, 6, 8% by weight). Two of the solutions were mixed by stirring magnetically, afterward sonicated in an ultrasonic bath in order to obtain a homogeneously mixed nanoparticle/epoxy solution. The glass and steel substrates were dipped into the solution at room temperature by utilizing mechanical dip coating equipment and withdrawn from the mixture solution at an optimum rate of 250 mm/min in order to enhance the uniformity of the films. The coated surfaces were cured at 175 °C for 30 min and kept in desiccators at room temperature before use. The thickness of the epoxy/alumina coatings was about 2–2.5 μm. Schematic representation of the preparation of superhydrophobic epoxy/alumina surfaces is given in Scheme 1. In order to evaluate the effect of the epoxy modification, control samples were also prepared without the addition of nanoparticles for each of the surfaces, herein named as “pure epoxy coating”. Additionally, glass and different steel substrates were coated with the purpose of verifying the suitability of the proposed superhydrophobic coating to different substrate materials.

### 2.3 Characterization of Surfaces

The contact angles of epoxy surfaces were measured by using the Attention Theta Lite contact angle meter apparatus. 5 μL droplets were used for measuring equilibrium contact angles of pure water and other liquids (tea, coffee, juice, milk, ethylene glycol, and glycerol). The advancing contact angles were measured by increasing droplet volumes from 3 to 8 μL, and the volume was withdrawn to 4 μL for receding contact angles. The contact angle hysteresis (CAH) was calculated as the difference between advancing and receding angles (CAH = θ_a_ – θ_r_). The contact angles were reported as the average of five independent measurements on each surface, and error bars are given in the plots to show the variations. The thickness of the films was measured by a Mitutoyo electronic digital micrometer at least at five points on each surface. The surface morphology and nanoparticle dispersion in the epoxy coating matrix were evaluated by SEM [FEI-QUANTA FEG 250-field emission scanning electron microscopy (FE-SEM)]. Atomic force microscopy (AFM) images were taken with ambient AFM (NanoMagnetic Instrument) by utilizing tapping mode with a commercial tapping silicon (Si) tip. The scan rate was 10 μm/s with a scan area of 2 μm × 2 μm. Three-dimensional (3D) AFM images and roughness parameters were calculated by using NanoMagnetics image analyzer software.

#### 2.3.1 Cross-Cut Adhesion Test

The adhesion between the coating and the substrates was evaluated by a cross-cut adhesion test, which was carried out according to the EN 13523-6 standard. The surface was drawn to form 25 squares perpendicular to each other with a cross-cut knife (TQC CC1000 adhesion test kit, 1 mm) that made 6 equal cuts. Adhesive tape with 10 ± 1 N adhesion strength on the scratched surface was applied to the surface for 1 min to ensure good adhesion. The tape was pulled from the surface at a speed of 0.5–1 s and at an angle of 60°. It was checked with a 10-fold magnifying glass whether the alumina-added epoxy coating was separated from the surface.

#### 2.3.2 Self-Cleaning Test

The self-cleaning behavior of superhydrophobic epoxy surfaces was evaluated using soil as contaminants on superhydrophobic epoxy surfaces. Water and other common liquids that are used in daily life (tea, coffee, juice, milk, ethylene glycol, and glycerol) were dropped onto the coated surfaces. The surfaces were also immersed in the liquids for 1 min and then taken out to observe the surface.

#### 2.3.3 Neutral Salt Spray Fog Test (Corrosion Test)

The test was carried out in accordance with the EN ISO 9227 standard. The saltwater solution was prepared using NaCl salt at a concentration of 5% (±0.5) and deionized water at 25 (±2) °C with a conductivity value not exceeding 20 μS/cm. The pH value of the saltwater solution sprayed into the cabinet was adjusted to be 6.5–7.2 at 25 (±2) °C by using sodium hydroxide (NaOH) or hydrochloric acid (HCl) in the salt water preparation tank of the device. All four sides of the samples were taped to prevent edge corrosion. The coated and uncoated samples were exposed to salt water fog at 35 (±2) °C in a closed cabinet for 48 h. After 48 h, the surfaces of the samples were checked and photographed. The corrosion resistance of the samples coated with coating solutions containing different Al_2_O_3_ nanoparticles was examined and compared.

## 3 Results and Discussion

### 3.1 Wettability and Contact Angle Results

**Figure 1:**
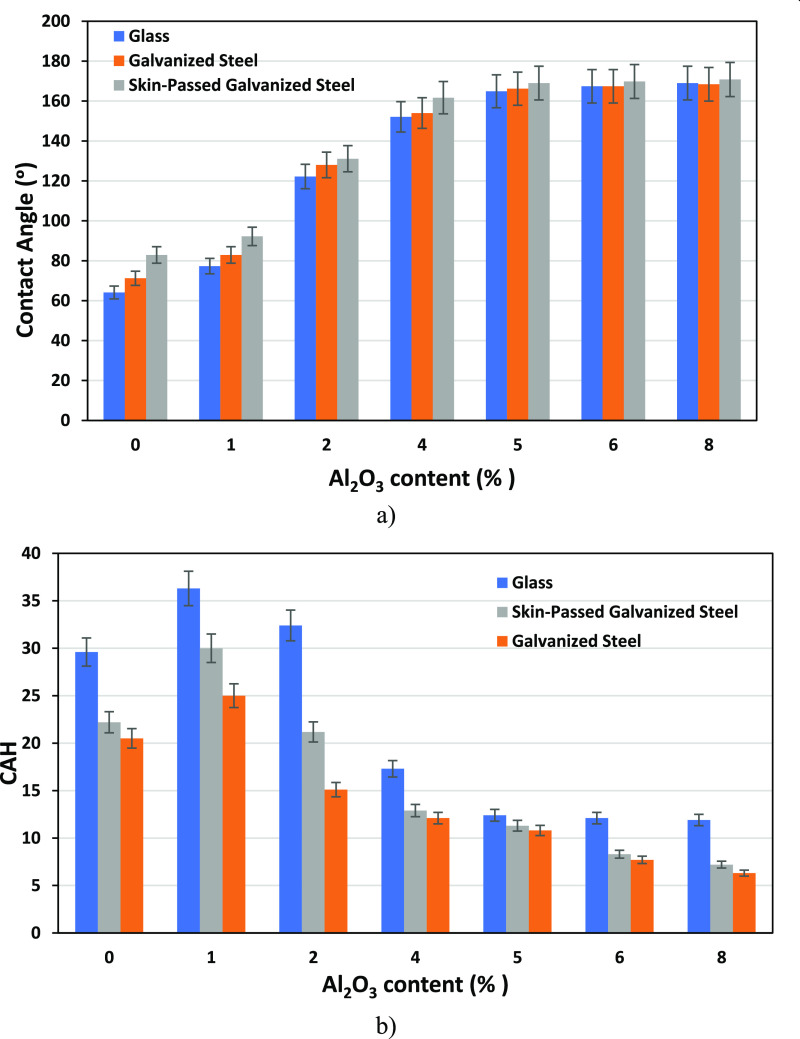
(a) Equilibrium water contact angle and (b) contact angle hysteresis (CAH) values of epoxy coatings as a function of Al_2_O_3_% concentrations on different surfaces.

The effects of alumina nanoparticle concentration on the wettability and surface morphology of epoxy coatings were investigated. Figure 1a shows the variation of equilibrium water contact angles as a function of alumina content on different surfaces. The contact angle of the epoxy-coated glass surface without nanoparticles was found to be 64.1° in accordance with the literature.^45−48^ By the addition of 1% Al_2_O_3_ nanoparticles into the epoxy matrix, the liquid repellency of the surfaces was poor, and the contact angle was only 77.3°. The nanoparticle concentration is the key parameter to achieve superhydrophobicity, as stated by Manoudis et al.^14,29^ They investigated the wettabilities of different polymer–nanoparticle composite systems by the spray coating method and concluded that the contact angle increased significantly with the increase of Al_2_O_3_ nanoparticle concentration as in concordance with our results.^14^ The critical particle concentration (CPC), which is the minimum particle concentration to obtain a superhydrophobic surface, is 4% Al_2_O_3_ with a contact angle of 152.1°.^14,19,49^ The highest superhydrophobic character, with a contact angle of 169°, was observed with maximum alumina content that is 8% wt. That is because the addition of nanoparticles could change the micro/nanostructure of the coating and the surfaces perform excellent superhydrophobic property at the higher nanoparticle contents.^19^ Hill et al. utilized isostearate functionalized Al_2_O_3_ nanoparticles to prepare superhydrophobic plastic surfaces by spray coating method. They reached 151° with the mass ratio of the nanoparticles: epoxy resin as 8.6:1.0.^28^ Penna et al. also observed superhydrophobic epoxy surfaces with alumina with high contact angles on the surfaces by using bilayer coating approach. They first brushed the stainless-steel substrate with epoxy and then spray coated with alumina dispersions with different solvents in two step.^18^ Wu et al. also prepared superhydrophobic alumina/epoxy coatings on aluminum plates. They used a two-step coating method, an inverse infusion process (IIP), by forming an uncured epoxy layer first on the substrate, then forming nanocomposite coating using air spraying method. In contrast to our study, the effect of particle concentration on different metal surfaces was not investigated, as a constant amount of alumina nanoparticles was dispersed to the epoxy solutions.^33^ It is an advantage that we could fabricate superhydrophobic surfaces via one-step coating procedure more easily and could achieve higher contact angles as 170°.

The contact angle of the epoxy-coated galvanized steel surface without nanoparticles was found to be 71.2°. It was observed that the contact angle increased significantly with the increase of nanoparticle concentration on all of the surfaces as in concordance with the literature.^14,15,19,29,49−51^ Superhydrophobic surfaces were obtained with an angle of 154° at 4% Al_2_O_3_, which is also a critical particle concentration here. In addition, it was observed that the equilibrium contact angle values were very close to each other at 5% Al_2_O_3_ and higher concentrations. Similarly, it was observed that the contact angle increased significantly with the increase of Al_2_O_3_ nanoparticle concentration on the skin-passed galvanized steel surface. The highest contact angles were obtained on skin-passed galvanized steel surfaces at all concentrations, as expected. The contact angle of the epoxy-coated skin-passed galvanized steel surface without nanoparticles was found to be 82.9°. Superhydrophobic surfaces were obtained with an angle of 161.7° at 4% Al_2_O_3_ concentration. The contact angle value reached to 170.8° at maximum alumina concentration. It was difficult to measure the contact angles on higher particle contents due to the droplets rolled off the surface toward the sides very easily and rarely stabilized (Supporting Information Video S1). The measurements were collected by carefully placing the droplets of water in a perfectly flat region of the sample.

The variation of the contact angle hysteresis (CAH) value with the change of Al_2_O_3_ concentration is given in Figure 1b. CAH was calculated by taking the difference between the advancing and receding contact angles (CAH = θ_a_ – θ_r_) and an important parameter to understand the movement of liquid droplets on solid surfaces. The Wenzel and Cassie–Baxter models are two classical models generally used for describing the wetting properties of superhydrophobic surfaces.^14,15,19,49,52−55^ The equilibrium contact angle generally increases with increasing nanoparticle concentration due to the increasing surface roughness, but CAH behaves differently and oppositely in the two models. When the drop generally sticks strongly to the surface and CAH is large, it is defined as Wenzel mode. When the liquid drop sits on the air pockets on the surface and slips easily from the surface, and the CAH is small then it is Cassie–Baxter mode.^14,15,49,54^ In this work, it was observed that CAH increased from 0% Al_2_O_3_ concentration to 1% Al_2_O_3_ concentration, besides it decreased with the increase of Al_2_O_3_ nanoparticle concentration on all surfaces. This situation showed that there was a transition behavior from the Wenzel model to the Cassie–Baxter model after from 1% Al_2_O_3_ concentration. A similar transition behavior was given by several authors in the literature.^14,15,49^ It was determined that the hysteresis values on glass, galvanized steel, and skin-passed galvanized steel surfaces were 9.9, 6.3, and 7.2 at 8% Al_2_O_3_ concentration, respectively.

### 3.2 Surface Morphology

**Figure 2:**
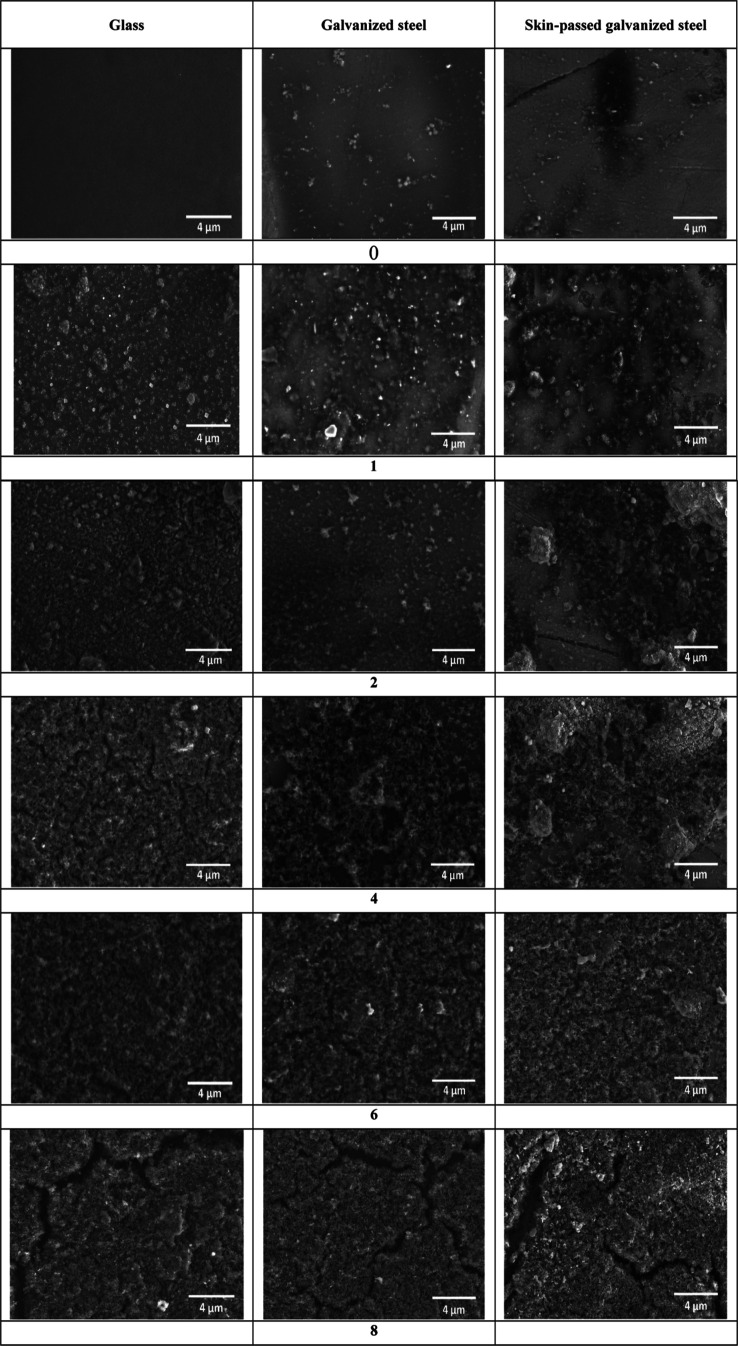
SEM photos of epoxy/alumina-coated glass, galvanized steel, and skin-passed galvanized steel surfaces with different alumina contents.

SEM photos of epoxy/alumina coated glass, galvanized steel, and skin-passed galvanized steel surfaces with different alumina contents are given in Figure 2. Pure epoxy coating without alumina particles was rather smooth, especially on glass substrates.^10,42,48,56−58^ At low contents of 1 and 2% alumina, there seen messy gaps on the surface. It was observed that when Al_2_O_3_ was added to epoxy surfaces, roughness was formed on the surface, and it increased as the concentration increased.^18^ Cai et al. also showed that the smooth morphology of the epoxy surfaces was enhanced with the addition of silica nanoparticles.^48^ As can be seen from the SEM images, especially at 4, 5, and 6% Al_2_O_3_ concentrations, the surfaces were completely covered with layer of nanoparticles, and the distribution of nanoparticles was homogeneous. In addition, surfaces with superhydrophobic properties were obtained starting from 4% Al_2_O_3_ contents. At higher concentrations, deeper cracks were seen on the surfaces.

The pure epoxy (non-Al_2_O_3_ nanoparticles) coated galvanized metal surface has a rougher and more lumpy appearance compared to the glass surface. Similar to glass surfaces, when Al_2_O_3_ was added, it was observed that the surface roughness increased, and with the increase of Al_2_O_3_ concentration, micro–nano roughnesses were obtained and the surface showed a porous morphology. Superhydrophobic behavior of a surface is a result of micro–nano roughness on the surface. With the roughening of the galvanized metal in the skin-pass unit and the addition of Al_2_O_3_ nanoparticles, a rougher structure was observed compared to the other (glass and galvanized steel) surfaces. In addition, deep cracks were observed on roughened galvanized metal surfaces at 8% Al_2_O_3_ concentration.

**Figure 3:**
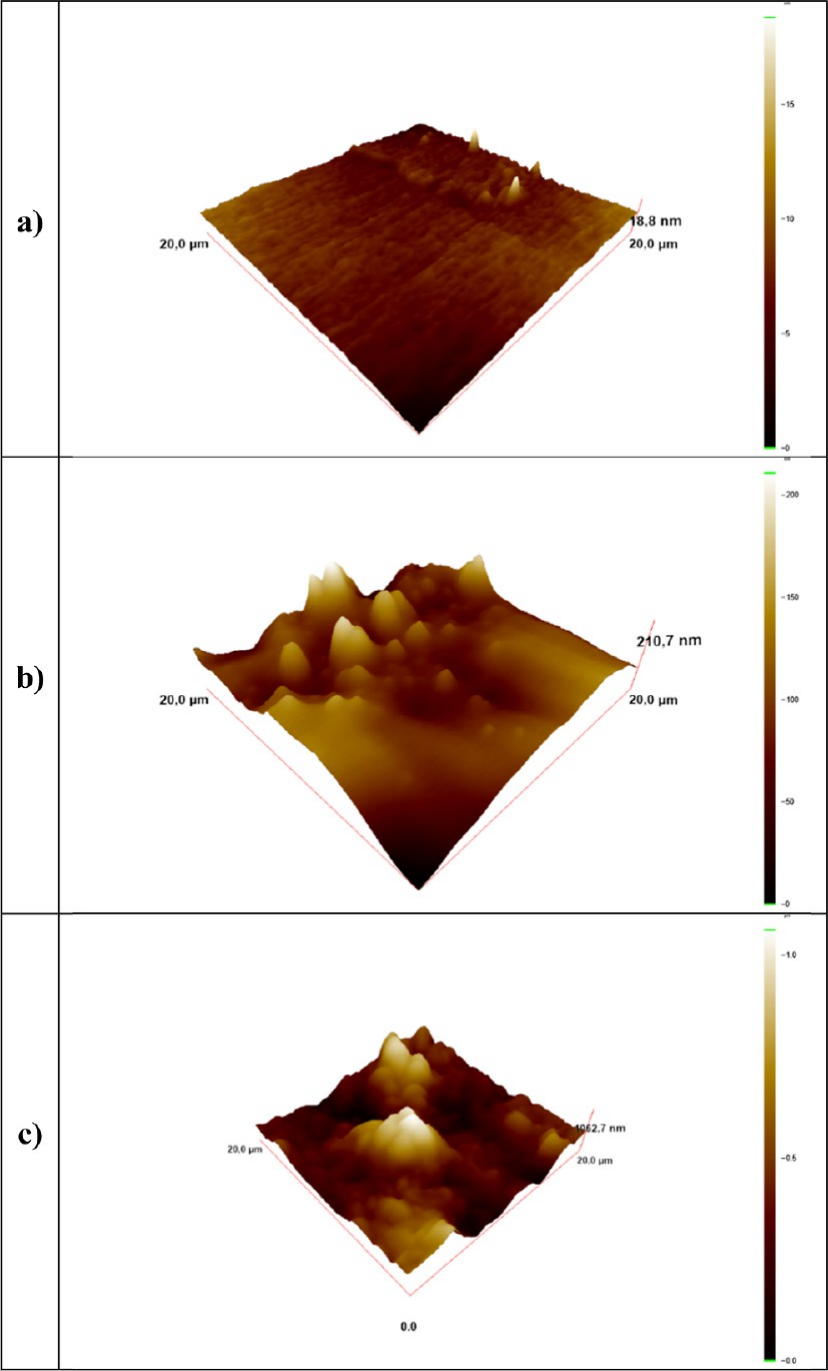
AFM images of (a) pure epoxy, (b) 1% and (c) 4% Al_2_O_3_-containing epoxy/Al_2_O_3_ coatings.

The AFM images of the epoxy surfaces prepared on a glass substrate from three different concentrations of alumina nanoparticle, which correspond to zero, low, and high concentrations for convenience of description, are given in Figure 3a–c. The pure epoxy surface is almost flat with a roughness of 1.15 nm, compatible with SEM images in Figure 2. In accordance with the literature, it was observed that peaks and valleys did not occur in the AFM images of the glass surface coated with only epoxy polymer.^59^ The roughness and low surface energy are indispensable for creating superhydrophobic surfaces. As the Al_2_O_3_ concentration increased, it was observed that hills and valleys were formed at a level that supported the roughness of the surfaces.

While the epoxy polymer had a flat surface in Figure 3a, the addition of Al_2_O_3_ nanoparticles caused roughness (Figure 3b,c). As the Al_2_O_3_ concentration increases, it is clearly seen that the roughness increases with the aggregation of Al_2_O_3_ nanoparticles. As a result of AFM analysis, the average roughness (Ra) values of three different glass surfaces having 0, 1, and 4% Al_2_O_3_ concentrations were 1.15 nm, 19.08 nm, and 0.15 μm, respectively. These results confirmed that the addition of Al_2_O_3_ nanoparticles to the epoxy polymer causes an increase in the roughness of the glass surfaces. Zhuang et al. showed the increasing roughness on epoxy surfaces by addition of silica particles with AFM images.^60^ Increasing the roughness increased the superhydrophobic performance by holding more air, consistent with the contact angle results.

### 3.3 Self-Cleaning Property of Superhydrophobic Epoxy/Alumina Surfaces

**Figure 4:**
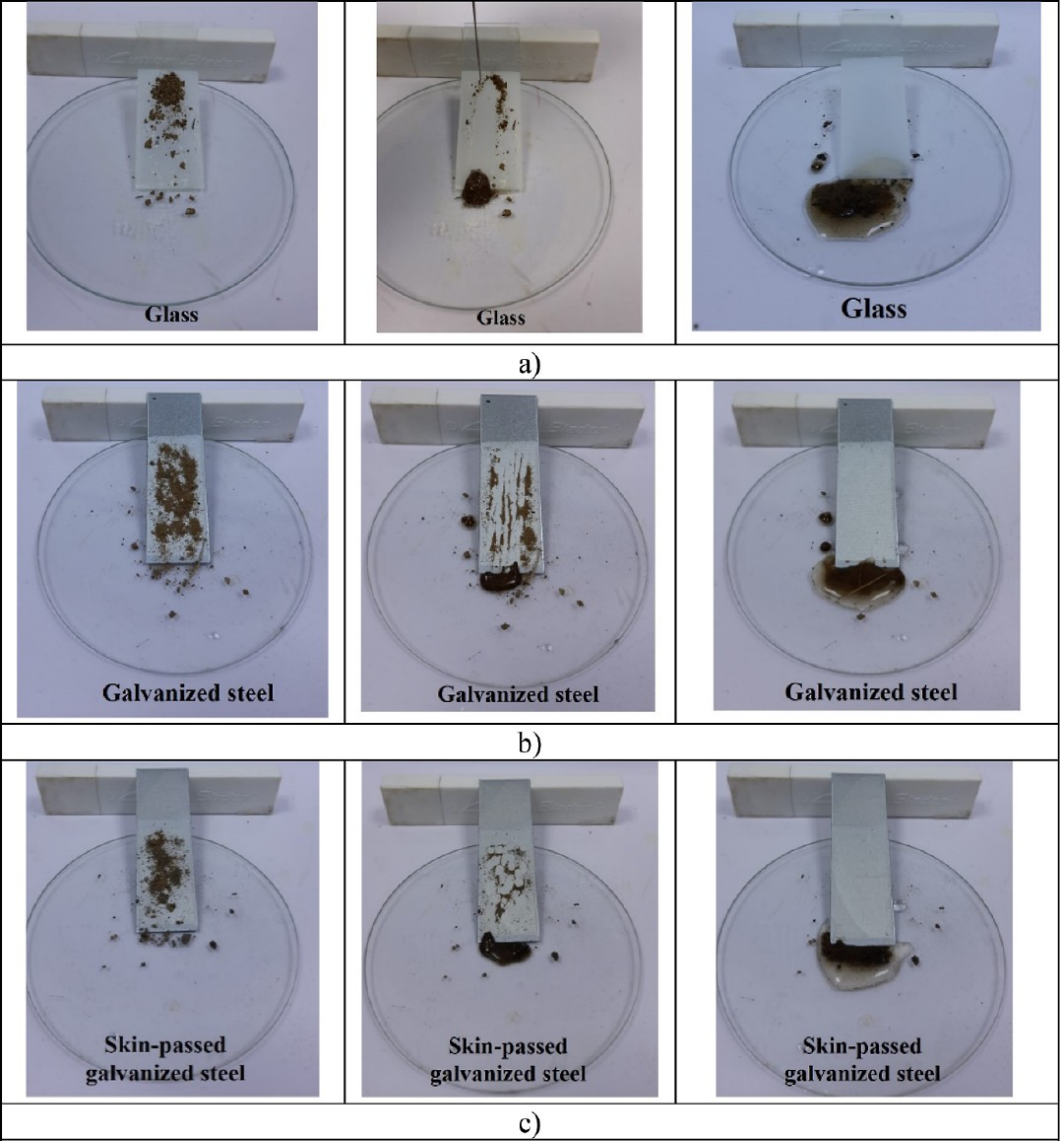
Self-cleaning behavior on (a) glass, (b) galvanized steel, and (c) skin-passed galvanized steel surfaces.

Self-cleaning performance is an indispensable property of superhydrophobic surfaces.^4,5,15,42,61^Figure 4 shows the self-cleaning process by dripping water with syringe on the surface of three different substrates coated with epoxy containing 5% Al_2_O_3_ concentration. The water was dropped on the surface slowly. It was observed that pollutants easily rolled away from the surface along with the rolling of water droplets. As the water droplets roll off, the dust on the superhydrophobic coating was carried away by the water droplets. The surface polluted by dust was cleaned in a short time, presenting the same clean surface state as before. Water droplets and dust on the surface of the uncoated surfaces hold and adhere to the surface. By comparison, the superhydrophobic coating has excellent self-cleaning properties (Supporting Information Video S2). Thus, in practical applications, the epoxy containing Al_2_O_3_ nanoparticles could efficiently protect the surfaces from pollutants. These properties made the epoxy with Al_2_O_3_ coating able to be applied to more practical applications, such as solar panels, exterior wall, automotive, oil pipeline, clothing, etc.^5^

**Figure 5:**
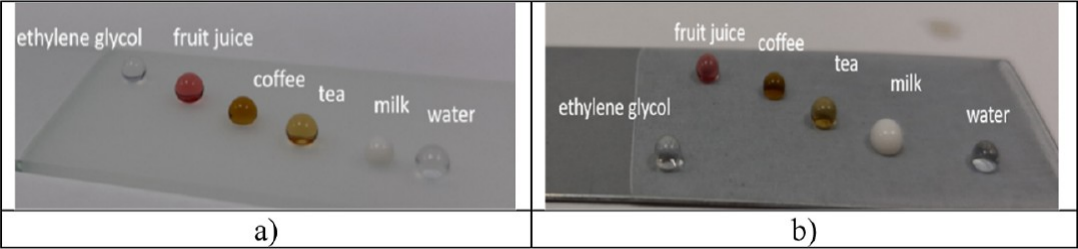
Contact angles of water and other common liquids on alumina-containing superhydrophobic epoxy surfaces coated on (a) glass, (b) skin-passed galvanized steel surfaces.

Figure 5 shows the contact angles of different everyday liquids on the surface of the substrates. These obtained superhydrophobic coatings have a high contact angle with the liquids, indicating that the surfaces have excellent antifouling properties.

**Figure 6:**
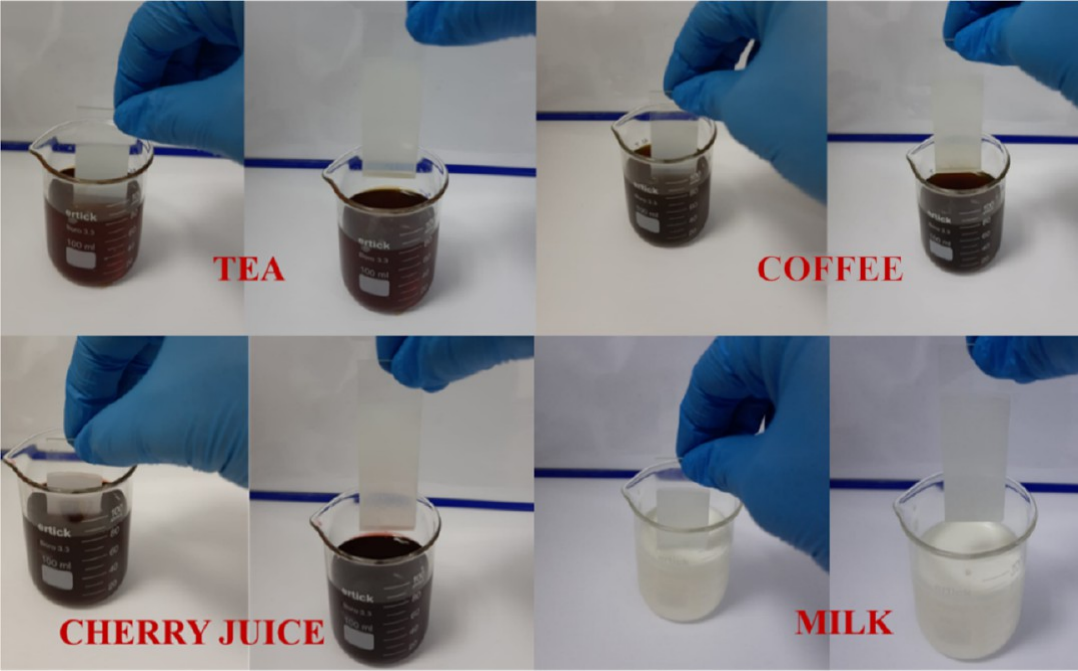
Repellency property of epoxy/alumina superhydrophobic surfaces prepared on glass substrates for common liquids: tea, coffee, cherry juice, and milk.

Figure 6 shows the repellent properties of epoxy/alumina superhydrophobic surfaces prepared on glass substrates for common liquids, including tea, coffee, cherry juice, and milk. The epoxy/alumina coatings were immersed in the liquids for 1 min, and no liquid drops were found on the coatings, showing that the obtained surfaces were difficult to be polluted by common liquids in daily life.

### 3.4 Adhesion Test

**Figure 7:**
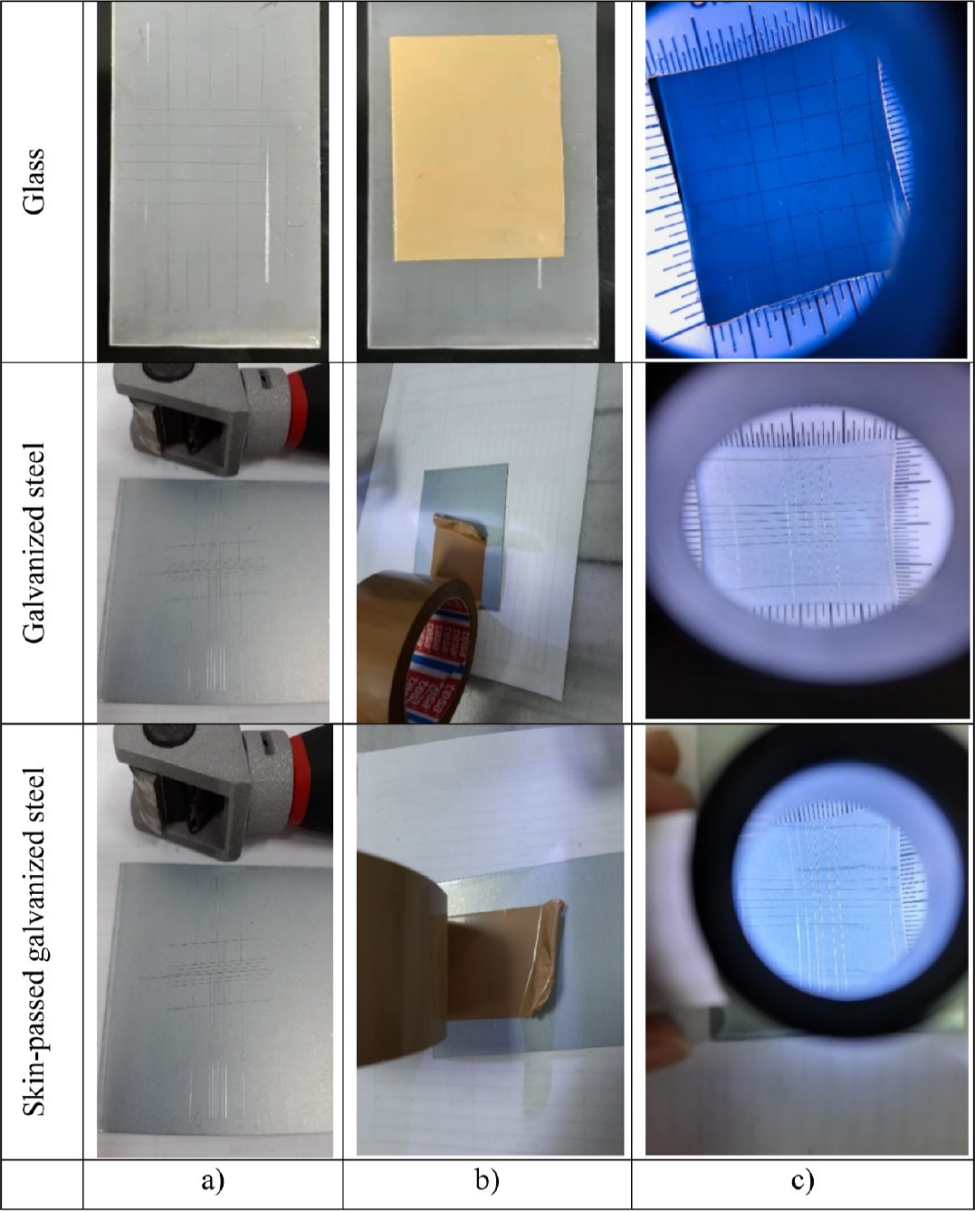
Photographs of the epoxy/alumina coatings (a,b) before and (c) after the cross-cut adhesion test.

To quantify the adhesion of the superhydrophobic coatings to glass or galvanized steel substrates, the cross-cut adhesion test was performed based on the EN 13523-6 Standard Test Method. Figure 7 shows photographs of the epoxy/alumina coatings before and after the cross-cut adhesion test for all surfaces. The surfaces after the cross-cut adhesion test showed no sign of any separation, and all the cuts were smooth. After peeling the tape from the surface, no significant difference was observed on the coating surfaces. The edges of the cuts were intact, and there was no coating detachment or delamination, clearly indicating the strong interfacial adhesion between the superhydrophobic epoxy/alumina coating and the substrate.^5,33,62^

The evaluation was calculated as a percentage by dividing the amount of rupture by the surface area, and the result of our study was defined as 0% loss of adhesion. The contact angles were measured as 114 ± 1° after tape was removed from the coatings on glass, galvanized steel, and skin-passed galvanized steel surfaces having a 4% Al_2_O_3_ concentration. Tape test results showed there occurred approximately 25% decrease on glass and galvanized steel surfaces, while 30% decrease observed on skin-passed galvanized steel surfaces. Especially, on the skin-passed galvanized steel surface, the process of the roughening of the surface increased the surface area of the surface, and the epoxy adhesion was even better; however, the removal of alumina after tape removal was larger due to the hills and valleys. As a result, since the films could be fabricated on the substrates in a one-step coating process and the resulting superhydrophobic glass surface was adequately robust, the prepared alumina-incorporated epoxy films might be widely used in the large-scale manufacture of superhydrophobic surfaces having self-cleaning properties.^63^

### 3.5 Corrosion Behavior of Epoxy/Alumina-Coated Steel Surfaces

**Figure 8:**
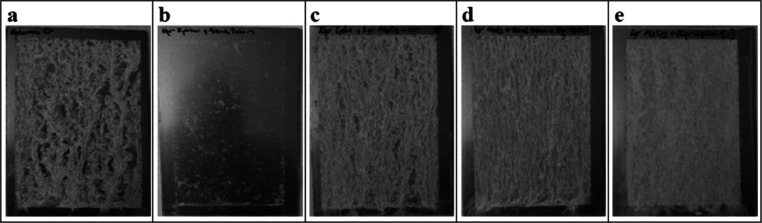
Photographs of galvanized steel surfaces (a) uncoated, (b) pure epoxy coated, (c) epoxy + 2% Al_2_O_3_, (d) epoxy + 4% Al_2_O_3_, (e) epoxy + 6% Al_2_O_3_ coated after a 48 h corrosion test.

The salt spray test is one of the important methods to determine the performance of metal surfaces in various environments.^4,10,36,39,64,65^ Cr(III)-based corrosion inhibitor passivation applied galvanized steel surfaces with equal amount of epoxy polymer were covered with coating solutions containing Al_2_O_3_ nanoparticles with different concentrations, and then they were put to the neutral salt spray fog test for 48 h. The photos, which were taken at the end of the corrosion test, are shown in Figure 8.

In the surface photos obtained after 48 h, white rust formation was observed on the uncoated galvanized steel surface as expected (Figure 8a).^35,37,66,67^ It is seen that only the epoxy-coated surface prevents corrosion on the surface to a large extent, since it largely prevents the contact of the galvanized steel against air and external factors.^64,68^ There is an insignificant amount of white rust and corrosion products on the surface. However, the epoxy coating itself has no self-cleaning effect with a low contact angle. On the Al_2_O_3_-added epoxy surfaces, the nanoparticles showed an anodic effect and caused the formation of white rust on the surfaces, but it was determined that red rust did not occur even though the alumina concentration was increased, indicating a good resistance of corrosion.^35^ It is known from the literature that the addition of nanoparticles to polymer systems provides significant barrier properties for corrosion protection.^39,44,64,69−72^ Similarly, it has been shown in the study of Sharifi Golru et al. that the addition of nanoalumina particles (2.5% by weight and 3.5% by weight) to epoxy caused an increase in the anti-corrosion property.^64^ Hamid et al. also obtained a good corrosion resistance after a salt spray test that there was about 95% white rust and no red rust occurred on the galvanized steel sample surfaces with the addition of 0.1 Sn wt %.^39^ The enhancement of corrosion resistance of the nanoparticle added coatings is attributed to specific properties of nanoparticles that have a smaller particle size and a large surface area.^64^ They ensure a passivation layer on the surface against environmental corrosion and also provide a self-cleaning and superhydrophobicity by increasing the surface roughness.^73^

The SEM images of the uncoated galvanized steel surface before and after corrosion are given in Supporting Information Figure S1. It was seen that the galvanized surface was deformed after corrosion, the zinc layer was eroded, and differences on surface elevation were gained, such as large valleys and hills. Figures S1c,d shows the SEM images of the pure epoxy-coated surfaces containing no nanoparticles before and after corrosion. It was seen that all surface was covered by epoxy, filling the gaps in the galvanized coating. Some white corrosion products or nodules were formed on the corroded surface. The SEM images of the surfaces coated with epoxy polymer containing 1% Al_2_O_3_ concentration before and after corrosion are given in Figures S1e,f. The homogeneous appearance, which was formed before corrosion, was deformed by the effect of corrosion. In addition, level differences occurred on the surface depending on the coating thickness.^36^Figures S1g,h shows SEM images of the surfaces coated with epoxy polymer containing 8% Al_2_O_3_ before and after corrosion. The deformation occurred by corrosion was greater and deeper than the surface with a 1% Al_2_O_3_ concentration. For this reason, it was observed that as the Al_2_O_3_ concentration increased on the surface, white rust occurred. No red rust was observed on any of the surfaces after 48 h. This supported that epoxy and Al_2_O_3_ nanoparticles acted as barriers on the galvanized steel surface.^64^

**Figure 9:**
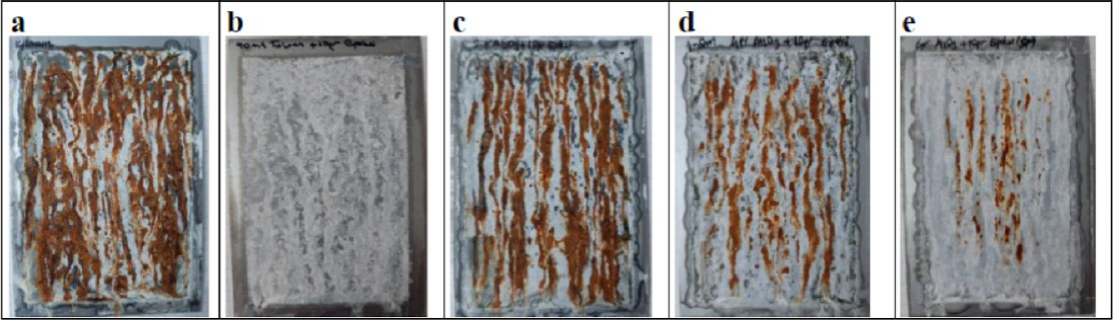
Photographs of skin-passed galvanized steel surfaces (a) uncoated, (b) pure epoxy coated, (c) epoxy + 2% Al_2_O_3_, (d) epoxy + 4% Al_2_O_3_, (e) epoxy + 6% Al_2_O_3_ coated after a 48 h corrosion test.

In Figure 9, red rust formation is observed on the surfaces due to the corrosion in a short time. On the roughened surface, the surface area expands, and thus the applied organic coating settles between the valleys and hills on the surface, providing an ideal adhesion for paint or organic coating. However, it decreased corrosion resistance by increasing surface deformation and area.^40^ In the surface images obtained after 48 h, an intense red rust formation was observed on the uncoated skin-passed galvanized surface. It was observed that only the epoxy coating prevented the formation of red rust but could not prevent the formation of white rust (Figure 9b). The addition of Al_2_O_3_ could not prevent the formation of red rust, with releasing the nanoparticles from the surface, the corrosion reaches to the metal surface giving red rust. It was seen that the amount of red rust decreased as the Al_2_O_3_ concentration increased (Figure 9d,e). It was observed that the formation of red rust was delayed with the addition of alumina nanoparticles to epoxy on skin-passed galvanized surfaces. It was achieved to have both superhydrophobic and anticorrosive surface properties together for outdoor metals in order to be used for longer times having self-cleaning ability.

## 4 Conclusions

In this study, superhydrophobic surfaces were obtained by adding hydrophobized Al_2_O_3_ nanoparticles at different concentrations to epoxy matrix by a simple, low-priced dip coating method. This fabrication method is one of the most common industrial methods that can be easily applied to almost any sized surface with high uniformity. The epoxy/alumina surfaces showed an excellent superhydrophobic surface property with a water contact angle of 170°. Superhydrophobicity of glass, galvanized steel, and skin-passed galvanized steel surfaces increased as the concentration increased. It also had self-cleaning property with very low contact angle hysteresis, showing a transition from the Wenzel state to the Cassie–Baxter state. In order to test the repellent properties of the surfaces, they were immersed in different liquids such as tea, milk, cherry juice, coffee. It was observed that the surfaces coming out of the liquids did not hold the drops. SEM images indicated that when Al_2_O_3_ was added to the epoxy surfaces, roughness occurred on all surfaces, and the surface roughness increased as the concentration increased. The increase in surface roughness due to Al_2_O_3_ concentration was supported by AFM analyzes on glass surfaces. Due to the imperfection and adhesion of the epoxy resin, the coating is very strong and has a high bonding force with the substrate. The corrosion resistance of the steel surfaces was examined by the neutral salt-water fog test. The corrosion resistance of the galvanized and skin-passed galvanized steel surfaces increased with the increase of Al_2_O_3_ nanoparticle concentration. It has been determined that although roughened galvanized surfaces have low corrosion resistance due to their nature, they significantly reduce the formation of red rust. All results indicated that, due to the simple preparation process and excellent performance, the epoxy/alumina-coated superhydrophobic glass and steel surfaces have wide application prospects.

## Supporting Information Available

Chemical composition of galvanized steel and skin-passed galvanized steel surfaces, SEM photos of galvanized steel surfaces before and after corrosion, and videos of rolled-off droplets and self-cleaning process on superhydrophobic surfaces (PDF)
Rolled off droplets on superhydrophobic galvanized steel surfaces (MP4)
Self-cleaning process on superhydrophobic galvanized steel surfaces (MP4)
