# Supplementary material for: Investigation of Superhydrophobic and Anticorrosive Epoxy Films with Al2O3 Nanoparticles on Different Surfaces
Source: ACS Omega. 2023 Jun 6;8(24):21559–70. doi: 10.1021/acsomega.3c00729 (PMC10286268; doi:10.1021/acsomega.3c00729)
Supplement: Supplementary file 1 — ao3c00729_si_001.pdf [file ao3c00729_si_001.pdf]

Supplementary Material

Investigation of Superhydrophobic and Anticorrosive Epoxy Films with Al_2O_3 Nanoparticles on Different Surfaces

Merve Dandan Doganci*[1,2], Hakan Sevinç[1]

[1] Polymer Science and Technology Graduate Program, Kocaeli University, Kocaeli, Turkey

[2]Department of Chemistry and Chemical Processing Tech., Kocaeli University, Kocaeli, Turkey

*Correspondence to:

M. Dandan Doganci, Department of Chemistry and Chemical Processing Tech., Kocaeli University, Kocaeli, Turkey

e-mail: merve.doganci@kocaeli.edu.tr

Table S1. Chemical composition of galvanized steel and skin-passed galvanized steel surfaces

Substrat	Steel Quality	%Cu	%Mn	%Ni	%Cr	%Al	%C	%Mo	%S	%Si	%P	%N	%V	%B	%Nb	%Ti
Galvanized Steell	DX51	0,180	0,160	0,060	0,030	0,030	0,020	0,010	0,007	0,020	0,006	0,007	0,002	0,003	0,001	0,000
Skin-Passed Galvanized Steell	DX51	0,330	0,200	0,110	0,080	0,025	0,058	0,023	0,014	0,013	0,009	0,008	0,002	0,001	0,001	0,001

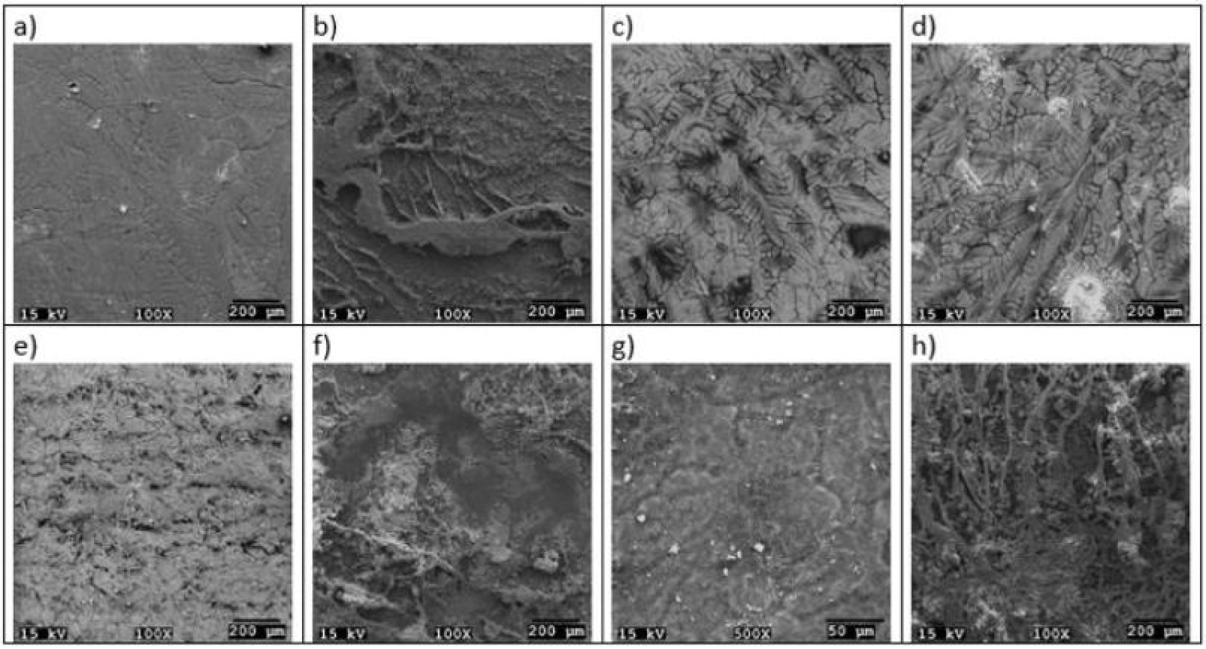

Figure S1. SEM photos of galvanized steel surfaces before and after corrosion a,b) uncoated c,d) epoxy coated e,f) epoxy+1 % Al_2O_3, g,h) epoxy+8% Al_2O_3

Video S1. shows the rolled off droplets on superhydrophobic galvanized steel surfaces

Video S2. is a self-cleaning process on superhydrophobic galvanized steel surfaces
